# Rule-Based Modeling of Chronic Disease Epidemiology: Elderly Depression as an Illustration

**DOI:** 10.1371/journal.pone.0041452

**Published:** 2012-08-28

**Authors:** Jean-Christophe Chiêm, Jean Macq, Niko Speybroeck

**Affiliations:** Université Catholique de Louvain (UCL), Institute of Health and Society (IRSS), Bruxelles, Belgium; Vanderbilt University, United States of America

## Abstract

**Background:**

Rule-based Modeling (RBM) is a computer simulation modeling methodology already used to model infectious diseases. Extending this technique to the assessment of chronic diseases, mixing quantitative and qualitative data appear to be a promising alternative to classical methods. Elderly depression reveals an important source of comorbidities. Yet, the intertwined relationship between late-life events and the social support of the elderly person remains difficult to capture. We illustrate the usefulness of RBM in modeling chronic diseases using the example of elderly depression in Belgium.

**Methods:**

We defined a conceptual framework of interactions between late-life events and social support impacting elderly depression. This conceptual framework was underpinned by experts' opinions elicited through a questionnaire. Several scenarios were implemented successively to better mimic the real population, and to explore a treatment effect and a socio-economic distinction. The simulated patterns of depression by age were compared with empirical patterns retrieved from the Belgian Health Interview Survey.

**Results:**

Simulations were run using different groupings of experts' opinions on the parameters. The results indicate that the conceptual framework can reflect a realistic evolution of the prevalence of depression. Indeed, simulations combining the opinions of well-selected experts and a treatment effect showed no significant difference with the empirical pattern.

**Conclusions:**

Our conceptual framework together with a quantification of parameters through elicited expert opinions improves the insights into possible dynamics driving elderly depression. While RBM does not require high-level skill in mathematics or computer programming, the whole implementation process provides a powerful tool to learn about complex chronic diseases, combining advantages of both quantitative and qualitative approaches.

## Introduction

Rule-based Modeling (RBM) is a computer simulation modeling technique relying on simple rules representing pieces of information that can integrate empirical data, existing knowledge and opinions [1]. These rules are translated into computer code, and the resulting program is then used to generate simulated data. The rules are then assessed by comparing the simulated data with observed trends. These rules can be, for example, “if-then” rules and do not need to be mathematical formulas. One of the most powerful features of RBM is its capacity to model complex phenomena in a simple and flexible way.

In epidemiology, RBM has been used to model the spread of infectious diseases. RBM is then specifically referred to as “Agent-Based Modeling” as rules are applied to individuals interacting together. The implementation of a rule-based model can integrate volatile human factors such as fear and irrationality. Detailed geographical maps can also be imported to mimic real environments. The introduction of this level of detail helps to recreate more realistic diffusion patterns. This methodology has already been strongly advocated as a tool in developing comprehensive containment strategies for worldwide pandemics [2]. The advances offered by RBM have encouraged researchers to consider using RBM in the modeling of chronic disease dynamics [3], [4]. RBM could be an alternative to overcome some weaknesses of more traditional approaches such as regression analysis.

In a chronic disease context, the use of quantitative analysis is complicated by the long timespan and the causal web that characterize chronic disease determinants. The long timespan between exposure and disease development requires long-term prospective data collection to properly identify risks and protective factors. This type of studies is costly and difficult. The causal web is made up of fuzzy factors such as context, social influence and life habits. Quantitative measurements of these types of factors rely on “social determinants” or “subjective health” indicators that have been criticized elsewhere [5], [6].

To compensate for a lack of quantitative data, or for the difficulty of utilizing such data, researchers typically use qualitative methods. However, although qualitative studies provide a powerful exploratory view, their results may appear too theoretical, too context-dependent, or even anecdotal. Regarding the flexibility of RBM, it reveals a promising technique, mixing both qualitative and quantitative information.

Among chronic diseases, elderly depression is a particularly challenging public health problem that could benefit from a simulation approach. In the next decades, the proportion of people older than 65 will increase globally. In the latter age group, depression is already considered a major health problem [7]–[9], as it affects functional capacities and causes other comorbidities. This will generate a potentially high economic and social burden on the whole society.

Studies have reported a significant association between depression and individual characteristics such as age, gender, low socio-economic status [10], bereavement [11], feelings of loneliness [12], increasing disability and cognitive impairment [13].

Two other key factors play a central role in elderly depression: social support, and specific events occurring in late life [14]–[16]. Firstly, the influence of social support on health status has already been proven to be correlated with emotional states such as feelings of loneliness [17], [18], mood [19], and depression [20]. However, social support is generally understood in an intuitive sense and it is hard to establish a proper definition that would encompass the different perspectives of support (perceived and enacted) [21]. Moreover, the measurement of social support requires a trade-off between the quality and quantity of social contacts [21]. Hence, while there is a need to further investigate the role of social support on health outcomes [21], [22], this concept is not easy to operationalize in studies [23]. Secondly, elderly depression has been associated with specific events of late life such as retirement, widowhood and institutionalization. These late-life circumstances affect, in turn, the social support of the elderly person [22]. Consequently, both these factors impact elderly depression with intertwined dynamics [16].

In this paper, an RBM approach is used to assess elderly depression in Belgium, formulating possible mechanisms of interactions between late-life events and social support. The primary purpose of the modeling exercise is to learn by interacting with experts and quantifying their opinions. This implementation may provide a powerful tool to learn from complex chronic diseases.

## Methods

Six main steps were used to design the RBM in this study:

1. Gathering empirical data
2. Building of a conceptual framework (a “theoretical mechanism”) for elderly depression focusing on social aspects
3. Elicitation of experts' opinions to quantify characteristics and behaviors
4. Design of scenarios
5. Translation of the rules into computer code
6. Comparison of the simulated data with the aforementioned empirical data.

### 1. Gathering Empirical Data

Data about depression in Belgium were available from “The Belgian Health Interview Survey - Interactive Analysis” (HIS) [24]. For each age, proportions of the population with symptoms of depressive disorder had been retrieved with confidence intervals (CI) of 95%. The indicator of depression used had been computed with the second item of the validated scale SCL-90-R, related to depressive disorder. This scale does not necessarily report a clinical diagnosis of depression, but considers the frequency, the intensity and the interaction of the symptoms of depression that can indicate the presence of such a condition (i.e. possible non-treated cases of depression were included) [25]. These data had been computed for 3 years (2001, 2004 and 2008). Data for individuals older than 92 years of age were not considered because the available sample sizes were smaller than 50. The following data (see in Appendix S5) were also retrieved from HIS, in order to better mimic the real population: 1) the gender proportions at age 65 in HIS (55.8% women), 2) the rates of depressed men (16.3%) and women (9.7%) at age 61, 3) the percentage of the population between ages 65–92 with reported depression in the past 12 months and who engaged in psychotherapy for this problem (16.9%), 4) the percentage of elderly persons between ages 65–92 and with income lower than 750 euros per month (11.5%).

In addition, the mortality rates of the population between ages 65 and 92 have been retrieved from the online site of the Belgian Federal Public Service of Economy [26]. Finally, the household composition retrieved from the Eurostat online database [27] reported that 67.73% of women were married at the age of 65 (see in Appendix S5).

### 2. Building of a Conceptual Framework

The development of a rule-based model requires the definition of some rules, simple by preference, which are believed to be the main drivers of the observed pattern. In the study at hand, the intertwined dynamics of the late-life course and social support are assumed to be the main driving forces of the general evolution of a pattern of depression. Other well-known factors of depression have been consciously set aside. The idea here is to learn by comparing this simplified representation of reality to observed depression patterns.

**Figure 1:**
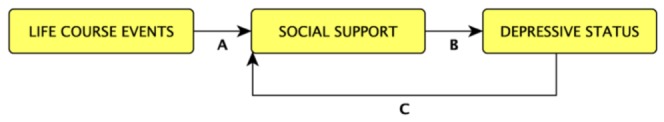
Conceptual framework. Events in the late-life events of the elderly person modify the social support they receive from different actors (A), which impacts their depressive status (B). In turn, this depressive status changes the social support they receive, creating a loop effect (C).

One possible conceptual framework to study the dynamics of elderly depression as a result of social support and late-life course is shown in Figure 1. It conceptualizes the idea that events in the late-life course of elderly people modify the social support they receive (A), which impacts their depressive status (B). In turn, this depressive status also changes the social support received, creating a loop effect (C). The different components used in the model are described as follows.

A **Events impact social support:** Each event in the late-life course of the elderly person influences the type of social support she/he receives. The Retirement [16] and the Death of the spouse [11] of the elderly person (widowhood) introduce a clear shift in the social support of the elderly person. The widowhood prompts the change of residence (Moving) for functional, economic or social reasons [28]. The Functional Decline of the elderly person induces a loss of autonomy [29], [30] impacting the social life of the elderly person. Finally, the Institutionalization [17] occurs, because the elderly person is too dependent in his activities of daily life, with subsequent social consequences.

For the purpose of this illustration, the first four events were considered as separated by five-year lags. A shorter lag of two years was considered between the Functional Decline and Institutionalization; the first being reported as a strong predictor of the latest [31], [32]. Hence these events were assumed to occur sequentially at the following average ages: 65, 70, 75, 80, 82.

Four types of actors were considered as involved in the social support of the elderly person [33]: 1) the Spouse [34], [35] (the person living in the same house as the elderly); 2) the Friends and Children (reported as having equal influence/importance for the elderly person [33]); 3) the professional Caregiver (for whom care is a professional activity); and 4) the Neighbors [36] (living in the neighborhood). The Loneliness (5) was considered as a special case of (the lack of) social support [17] and is considered as a ‘source’ of social support by extension. The relative importance of these five sources of social support of the elderly will be further designated as the social landscape of an elderly person.

B **Social support impacts the depressive status:** Three states of depressive status (DS) were used and represented by specific numerical values: Non-depressive (+1); Neutral (0); and Depressive (−1). These three states allowed the study to overcome a dualist model of mental disease where absence of specific issues is equated with positive mental health [37]. The Non-depressive state refers to a positive state of mental health (well-being). The Neutral state refers to an intermediate case where possible anxiety and psychological stress may occur, but do not reveal chronic pathology. In the Depressive state, the observed depressive symptoms are sufficient to require therapy if diagnosed. In addition, the use of the Neutral state indicates a less versatile effect of time. Indeed, between +1 and −1 there is a Neutral transition state, which reflects better the chronic (long-term) condition.

Each source of social support has an impact on the DS of the elderly person. Three possible types of impact on the DS were considered: Positive (+1) (the contact improves the DS); Neutral (0) (no impact on the DS) and Negative (−1) (the contact deteriorates the DS).

At each time step, these impacts were added to the current DS to compute a new DS. The DS values were limited to values between +1 and −1 (e.g. if the DS value exceeds +1, it is set back to +1).

C **Link between the depressive status and the social support:** The DS of the elderly person influences his contacts with his social landscape, as represented by loop C. This effect was modeled by using two different relations (A), based on the DS of the elderly person. One specific social landscape was defined for elderly people with Non-depressive and Neutral DS (+1 or 0). Another specific social landscape was used for elderly people with Depressive DS (−1).

Consequently, depending on the DS of the elderly, one of the two representations of the social landscape was applied.

### 3. Elicitation of Experts' Opinions

The conceptual framework allowed for the development of a questionnaire with questions related to the parameters needed in the model. The questionnaire (shown in Appendix S1 (English) and Appendix S2 (French)) was presented to experts to elicit a quantified opinion of the elderly depression parameters used in the model.

**Figure 2:**
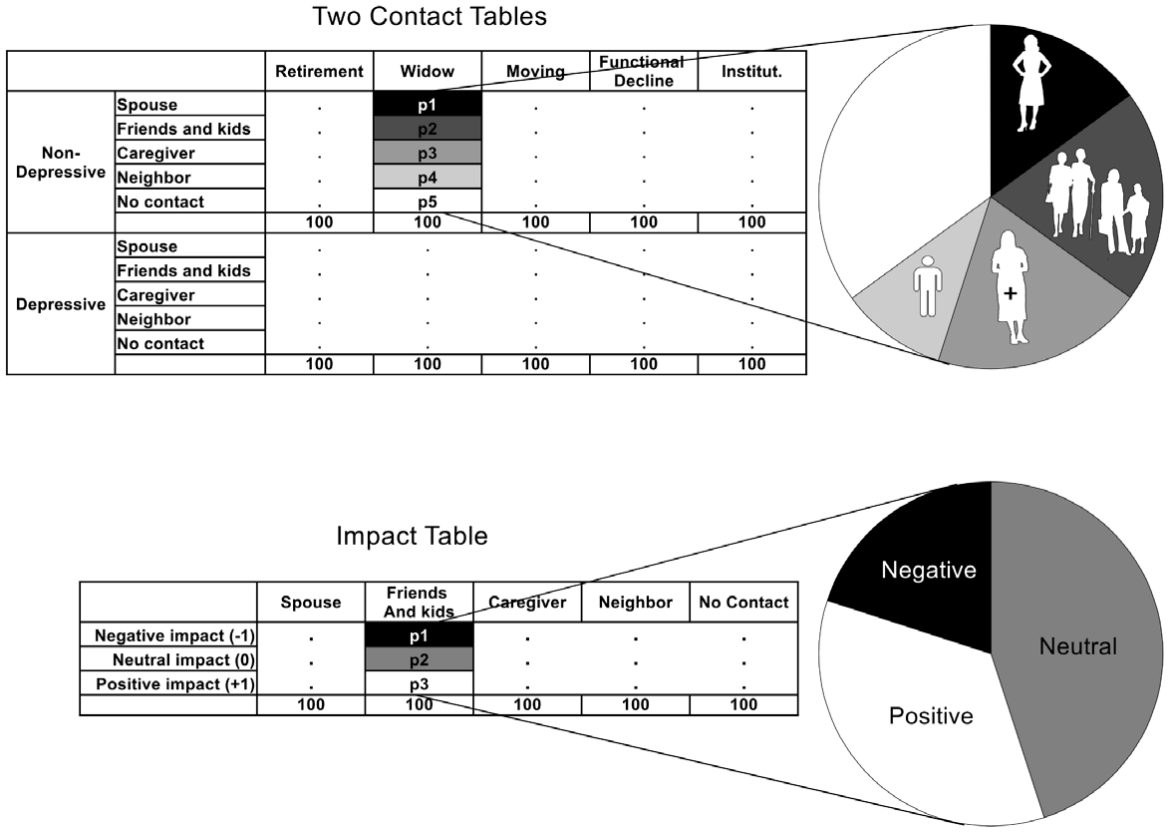
Questionnaire description. Two Contact Tables, one Impact Table as presented to the experts for elicitation. A graphical interpretation with pie plot was used to visualize the proportions.

The questionnaire is made up of three tables (rows×columns) that the experts were asked to fill out. The structure of these tables is provided in Figure 2 together with graphical representations. Each column entry in the tables defines the relative proportion in the corresponding pie chart. The tables are defined as:

- Two “Contact” Tables (5×5) characterizing the relative importance of each source of social support (row) at each key late-life event (column). The concept of “importance” was taken at large, considering not only the frequency of contacts, but also the intensity of the social support, as perceived by the elderly person. The two tables differentiate contacts for non-depressive and depressive elderly persons.
- One “Impact” Table (3×5) characterizing the probabilities of positive, negative, or neutral impacts (row) resulting from contacts with the sources of social support (column).

The questionnaire was integrated within an Excel sheet where percentages were automatically plotted within pie charts. This helped the experts to visualize their answers. The respondents were also asked to report their age, occupation, number of years of experience in this occupation, and number of contacts with people older than 65 years in the preceding week.

The questionnaire was submitted to Medical, Pharmacy and Public Health students of the Université Catholique de Louvain-la-Neuve (UCL). Among the students in the Master in Public Health program, many were already health care professionals such as doctors and nurses. Professional researchers in the field of elderly care of the Institute of Health and Society (IRSS/UCL) were asked to fill out the questionnaire as well.

This research was not a clinical trial. Only field experts were asked to answer a questionnaire. This questionnaire did not violate their privacy. Only their opinion was elicited, and their knowledge ‘quantified’. Due to this context, no institutional review board or ethics committee was approached and neither informed consent nor ethics approval was requested from the experts. The data were anonymized, and the field experts were aware that their answers were going to be used in a research paper.

### 4. Design of Scenarios

The flexibility of RBM allows for the conception of multiple scenarios. A simple scenario was used as a starting point. Rules were then added successively in order to better mimic the real population, investigate a possible treatment effect and explore the effect of socio-economic status (SES) on the elderly depressive state. These scenarios were implemented only to illustrate the usefulness of the method.

The first simple scenario investigated the experts' answers regarding the conceptual framework. While the focus was only on the ages between 65 and 92, individuals of 61 years old were created and their DS was initially neutral. This allowed for running the individual process to obtain a population at 65 randomized regarding the DS. They were considered as married but not within the same population. A personal sequence of ages for the events was generated, around ages (65, 70, 75, 80, 82) with a random normal noise with standard deviation (stdv) of 2, for illustrative purposes. After the age of 65, individuals were removed from the population at random, following the empirical mortality rates by age [26].

In the second scenario, a more realistic population was created to include the effects of gender (55.8% women [24]). In addition, the population created at 61 mimics a population with 16.3% of depressed women [24] and 9.7% of depressed men [24]. Three marital statuses were considered: single, married and widow. The women were married to men, 1 or 2 years older in order to reflect the mean empirical lags between the ages of wives and husbands (0.93–1.5 years) [38]. Therefore, 67.73% [27] of 61-year-old women were married to men of 62 or 63 years old. The remaining women and men were set to single. The specific effect of widowhood on the DS was modeled as follows. On the one hand, single elderly individuals were treated as if they never had a spouse. Both their Contact Tables were then updated without spousal influence and preserving the relative importance of other sources of social support. On the other hand, married elderly individuals were bound to each other. While they were both alive they remained together in the age period before widowhood.

When one of the spouses died, the marital status of the remaining spouse was set to widow and his/her age of widowhood was set to their current age. If this age of widowhood was lower than the expected age (< = 70), the ages for the subsequent events were set so as to fit the usual ages. But if widowhood occurred later, the three remaining events were spread out till the end of life preserving time lags between events after widowhood (+5, +5, +2). In addition, the DS of the widow was set to Depressive for one year, as widows are reported to experience depressive episodes in the first year of widowhood [39].

The third scenario investigated the possible effect of a treatment for depression, namely psychotherapy. The percentage of depressed persons treated by psychotherapy (16.9% [24]) were selected each year over the whole population. Their DS was improved to a Neutral DS (0) for half of them and to a Positive DS (+1) for the other half, for illustrative purposes.

Finally, the fourth scenario considered a distinction regarding the socio-economic status (SES) of the elderly. The percentage of elderly persons between 65 and 92 with income lower than 750 euros per month (11.5% [24]) was used as a raw index of socio-economic distress. For illustrative purposes, one hypothesis that could be tested was that these disadvantaged people had greater chances to experience social contacts resulting in a negative impact on their DS. Therefore, a new Impact Table was computed for these disadvantaged elderly persons, where the relative chances of negative impacts were increased. Hence, for all sources of social support in the Impact Table, a quarter of the values of both positive and neutral impacts were transferred onto the negative impact.

### 5. Translation of the Rules into Computer Code

The translation of the rules into computer code should be seen primarily as a formalization of the conceptual framework integrating the experts' opinions. A schematic representation of the algorithm is first pictured to illustrate the subsequent formal algorithm.

**Figure 3:**
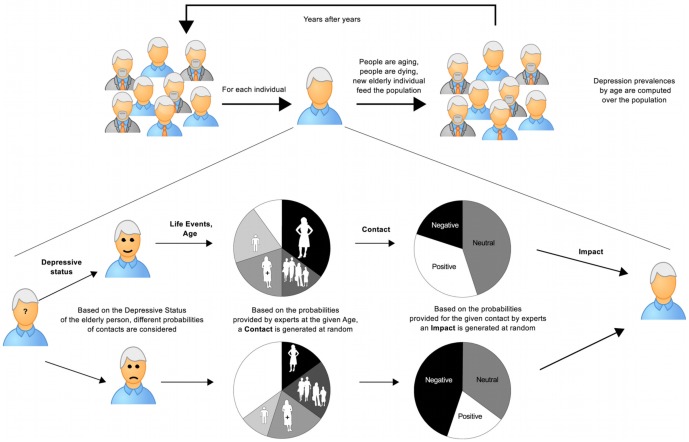
Schematic algorithm. A population is simulated where each individual elderly person is processed. Elderly depression dynamics are influenced by late-life events, social support and personal depressive status, and this influence is quantified by experts' opinions. Each year some elderly people die and new ones are integrated in the population.

A **Schematic algorithm:** The conceptual framework induces a dynamic of elderly depression in a population that can be schematically described as in Figure 3. Given a population, each individual is processed year after year. Based on the Depressive Status of the elderly person, different probabilities of contacts are considered. Based on the probabilities provided by experts at the given Age, a Contact is generated at random. Based on the probabilities provided for the given Contact by experts, an Impact is generated at random. Each year people are aging, people are dying; new elderly individuals are added to the population.

At the end of the simulation depression prevalences by age are computed over the population.

**Figure 4:**
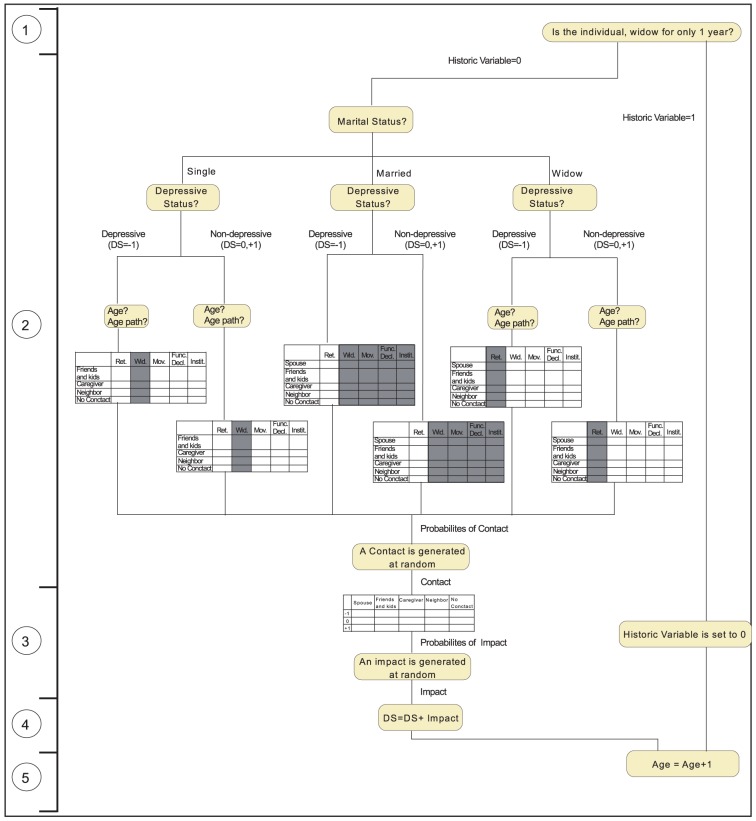
Formal Algorithm at Individual Level. 1) The Historic Variable is checked to see if the individual is widow for only one year; if this is the case, the new widow processes directly step 5. 2) Decision tree leading to the random generation of a Contact with one of the actors (grayed columns of the tables are not used. 3) An Impact is generated at random. 4) This impact is added to the current Depressive State 5) The Age is incremented.

**Figure 5:**
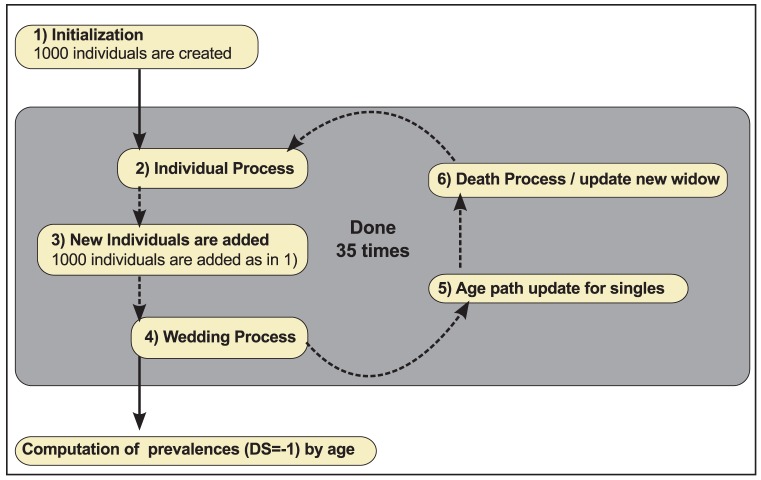
Formal algorithm at population level. 1) An artificial population of 1000 individuals is initialized. 2) The Individual process is run for each individual. 3) New individuals are added. 4) Individuals are married. 5) The Age path of singles is updated. 6) Individuals die and their corresponding new widows are created. For one simulation, steps 2–6 are performed 35 times. At the end of the simulation, the prevalences of depressed individuals are computed by age.

B **Formal algorithm:** The implementation of the second scenario is described here, only to illustrate the formal writing of the rules represented in Figures 4 and 5. The algorithms have been implemented in Matlab R2011a. The source codes together with installation and user instructions are provided in the Supporting Information (Appendix S6 and Appendix S7). These can also be downloaded from the following link https://sourceforge.net/projects/rbmelddep/.

Prior to the simulation, some additional data need to be computed. First, based on a virtual population of 1000 individuals at age 65, the number of individuals to remove between 66 and 92 of age was computed in order to fit the empirical mortality rates.

Then, for singles, who do not have a spouse, a specific Contact Table is computed. The probabilities in the Contact Tables are adjusted so that the spousal contact disappears: the probabilities of the four other contacts are reweighted to constitute 100% of the social landscape.

The algorithm at individual level is described in Figure 4. Each individual goes through an iterative process, with one iteration representing one year. Each individual can be characterized through ten variables: Age, DS, Age Path (i.e. five variables indicating ages of life events), Gender, Marital Status, and a link with the corresponding spouse (if married). For implementation purposes, a Historic Variable is created to indicate whether a widow is in the first year of widowhood (1 indicates that this is true). The following steps are performed:

1. If the Historic Variable is equal to 1, indicating that the individual has been widowed for only one year, this Historic Variable is set back to 0. The depressed state (DS = −1) is preserved translating the depression subsequent to the widowhood.
2. Depending on the Marital Status, the DS, the current Age, and the Age Path of the elderly individual, the column with the relative contact probabilities is retrieved from the corresponding Contact Table. Based on these relative probabilities a Contact with a source of social support is selected at random. Note that the first percentage valid for retirement is already applied before the Age of retirement; the last percentage valid for institutionalization is applied until the end of life.
3. Based on the selected Contact, the column with the relative impact probabilities is retrieved from of the Impact Table. Based on these relative probabilities an Impact on the DS is selected at random.
4. The selected Impact is added to the current DS. When values of +2 and −2 for DS are reached, they are adjusted to +1 and −1 respectively.
5. The Age is incremented by one year.

The algorithm at population level is described in Figure 5. The artificial population undergoes the following steps:

1. At the beginning of the simulation, an initial population is created with 1000 elderly individuals with Age = 61. They are single and their gender is assigned randomly with 55.8% women. Their DS is set to Depressive (DS = −1) for women (16.3%) and men (9.7%); otherwise their DS is set at random to Neutral or Non-depressed with equal probability. Their age of retirement is set around mean 65 with a normal noise (stdv = 2).
2. The individual process is run for each individual in the population.
3. New elderly individuals are added to the population as in Step 1.
4. Once men of 62 and 63 years old are available, 67.73% of the new 61 years old women are assigned as married to them. At 64 the Marital Status of each man and woman is either single or married.
5. After 63, the singles will stay single until the end of their lives and their age path can be generated. Singles do not experience widowhood. Random Ages are generated for moving, functional decline and institutionalization with a normal distribution around mean ages (75, 80, and 82) and with a standard deviation of 2. The sequence of events is preserved so that if the randomization inverts ages for events, they are reverted with only one year of lag. For example if the Age for functional decline is randomly set at 72 before the Age of moving at 74, then the Age of functional decline is set back to 75.
6. Once individuals reach age 65, they start to die. A pre-computed fixed number of individuals are removed at random for each age to fit the empirical mortality rates. Their marital status is checked. If they are widows or singles, no further step is taken. If they are married, 1) their spouse turns widow, 2) their spouse's age of widowhood is set to the current age 3) their spouse's DS is set to −1 to represent the year of depression and 4) Historic Variable is set to 1 and 5) the rest of the spouse's age path is updated till the end of life. If Widowhood occurs before 70, the normal mean Ages for moving (75), functional decline (80) and institutionalization (82) are preserved. Otherwise, the 3 remaining events are scattered in between widowhood and the end of life, preserving the time lags between events after widowhood (age of widowhood +5, +5, +2 years, respectively). This age assignment strategy is set for illustrative purposes only.

This algorithm is run for 35 iterations to obtain a representative population at each age between 65 and 92. Indeed, in the initiation of the wedding process, the very first women could not get married because no men of 62 or 63 were available. Thirty-five iterations are then required to allow them to be removed. At the end of the simulation, the prevalences of depressed individuals (DS = −1) are computed by age.

For each scenario, 50 simulations are run and the mean is computed over the 50 final depression prevalences per age. This averaging process is used to reach equilibrium in population age and characteristics.

### 6. Comparison with the Empirical Data

The generated simulated data were plotted against the empirical prevalences together with their intervals of confidence, allowing a graphical inspection of the fit between the two patterns. The number of times the simulation estimates fell within the empirical confidence interval was used as a first numerical criterion to compare the curves.

In addition, the Wilcoxon Signed-Rank test for the nullity of the difference between the paired two-samples was run to test the fit between the longitudinal patterns of the empirical and simulated curves.

Finally, the simulated data were also compared with the regression line over the empirical data and their correlation coefficient was computed. The two samples' Kolmogorov-Smirnov statistics was also computed to test whether the two samples could come from the same distribution.

## Results

**Figure 6:**
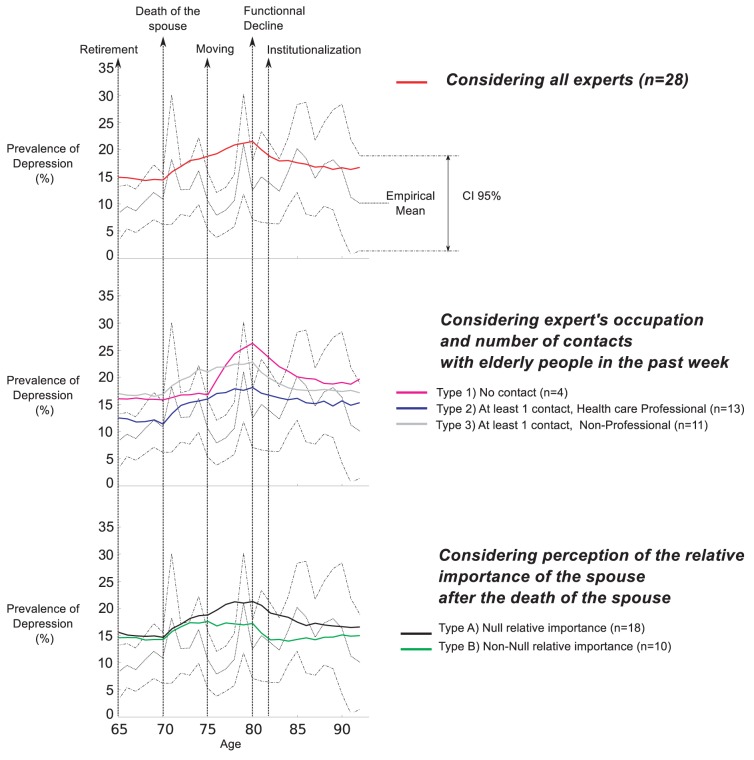
Simulated prevalences by typology (Scenario 1). The Scenario1 reflects simply the expert's answers. Simulated patterns of elderly depression prevalence by age are generated based on experts' answers, considering several typologies of experts. Empirical Mean and CI 95% of depression prevalence, retrieved from the Belgian Health Interview Survey, are plotted for comparison.

**Figure 7:**
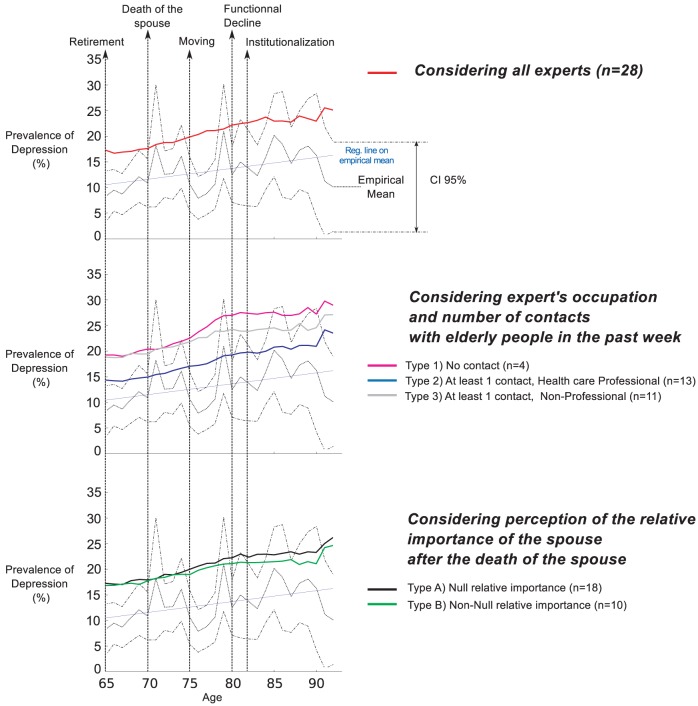
Simulated prevalences by typology (Scenario 2). The Scenario 2 introduces a wedding between husband and wives and defines 3 marital statuses: single, married and widow. Simulated patterns of elderly depression prevalence by age are generated based on experts' answers, considering several typologies of experts. Empirical Mean and CI 95% of depression prevalence were retrieved from the Belgian Health Interview Survey. They are plotted for comparison together with the regression line on the empirical prevalence.

**Figure 8:**
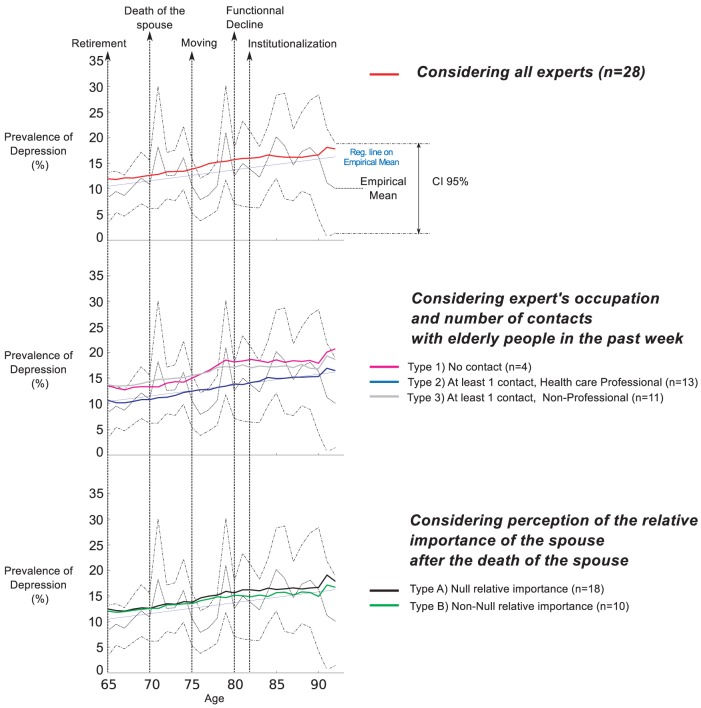
Simulated prevalences by typology (Scenario 3). The Scenario 3 introduces the effect of a treatment by psychotherapy. Simulated patterns of elderly depression prevalence by age are generated based on experts' answers, considering several typologies of experts. Empirical Mean and CI 95% of depression prevalence were retrieved from the Belgian Health Interview Survey. They are plotted for comparison together with the regression line on the empirical prevalence.

**Figure 9:**
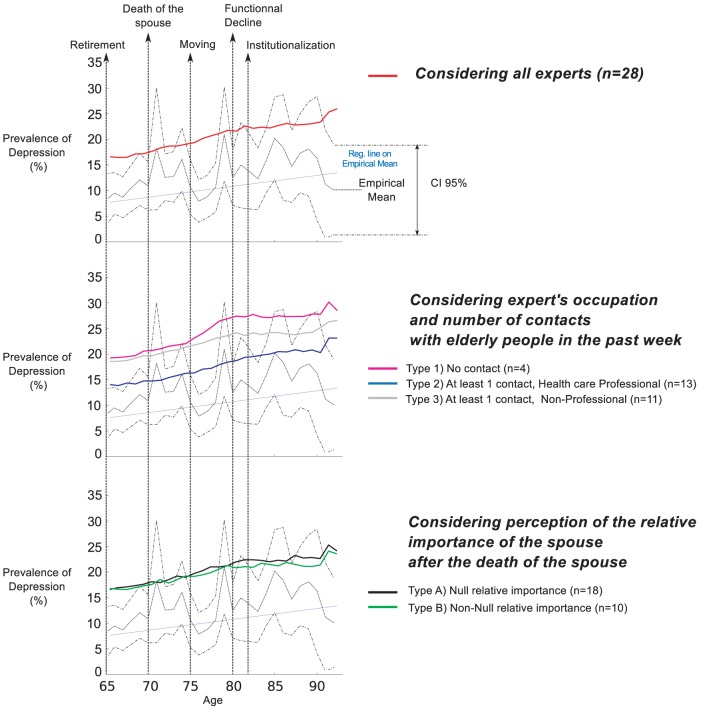
Simulated prevalences by typology (Scenario 4). The Scenario 4 introduces a distinction regarding the socio-economic status. Simulated patterns of elderly depression prevalence by age are generated based on experts' answers, considering several typologies of experts. Empirical Mean and CI 95% of depression prevalence were retrieved from the Belgian Health Interview Survey. They are plotted for comparison together with the regression line on the empirical prevalence.

Empirical average depression levels and confidence intervals retrieved from “The Belgian Health Interview Survey” are plotted with dotted lines in all panels of Figures 6–9. The regression line over the empirical prevalences is also plotted in Figures 7–9.

### 1. Experts Sampling

In total, 200 questionnaires were sent or requested and 28 questionnaires were filled out: 7 with supervision and accompanying oral clarification, and 21 received by mail.

Some respondents commented spontaneously that the questionnaire was surprising at first, but easy to complete “once the principle had been understood.” Mean Contact and Impact Tables, averaged over their answers, are provided in the Supporting Information in Appendix S3.

### 2. Categories of Experts

Two criteria based on the profile and the answers of the respondents were used to define two typologies (classifications) of experts.

Firstly, the experts were classified by their number of contacts with elderly people in the past week and their occupation:

- Type 1 (n = 4) were experts with no elderly contact during the past week.
- Type 2 (n = 13) were health care professionals with at least one contact with one elderly person during the past week.
- Type 3 (n = 11) were experts with at least one contact with one elderly person during the past week but who did not have an occupation related to health care.

Secondly, the experts were dichotomized according to their answers in the Contact Tables:

- Type A (n = 18) attributed a strictly null relative importance of the spouse ( = 0%) in the social landscape after the death of the spouse.
- Type B (n = 10) attributed some relative importance of the spouse (>0%) in the social landscape, even after the death of the spouse.

The Type A respondents attributed a null influence for all three events following the spouse's death, for Non-depressive as well as for Depressive cases. Note also that for the Type B respondents, influence of the spouse after the death was non-negligible, as it ranged from 10% to 55% over all three events following the spouse's death.

The answers of the experts matching the aforementioned Types were averaged and used in simulations (see in Appendix S3). The interquartile range of each entry in the Contact and Impact Tables was computed to provide an indication of the agreement between experts (see in Appendix S4).

### 3. Simulated Results

Simulations were run for each scenario using the Contact and Impact Tables averaged first over all experts and then over experts matching the aforementioned typology definitions. The resulting prevalence evolutions for these scenarios are shown in Figures 6–9, and statistical and numerical indices are reported in Table 1.

**Table 1:** Statistical and numerical tests.

Type of experts	Occurrences within CI	WSR	K-S	r
**Scenario 1**				
Type All	19	0.0001	0.75	0.3186
Type 1	14	0	0.9286	0.482
Type 2	23	0.0272	0.5	0.5529
Type 3	15	0	1	−0.0029
Type A	18	0.0001	0.7857	0.2698
Type B	21	0.019	0.6071	−0.1711
**Scenario 2**				
Type All	9	0	1	0.9734
Type 1	6	0	1	0.9509
Type 2	17	0	0.7143	0.9813
Type 3	7	0	1	0.9561
Type A	9	0	1	0.9738
Type B	11	0	1	0.9433
**Scenario 3**				
Type All	26	0.0382	0.3571	0.9741
Type 1	20	0.0004	0.5714	0.9354
Type 2	27	0.9455*	0.1429*	0.9869
Type 3	22	0.0008	0.5714	0.9312
Type A	26	0.0272	0.3929	0.9659
Type B	26	0.145*	0.25*	0.9596
**Scenario 4**				
Type All	7	0	1	0.9787
Type 1	2	0	1	0.9498
Type 2	9	0	1	0.9836
Type 3	6	0	1	0.9648
Type A	7	0	1	0.9707
Type B	8	0	1	0.9502

Statistical and numerical indices of the comparison between the simulated and empirical data, by scenario and typologies of experts (1st column). The 2nd column indicates the number of times the simulation estimates falls within the empirical confidence interval (CI). The 3rd column indicates the Wilcoxon Signed Rank statistic (WSR). Cases reported with an ‘*’ indicate that the nullity of the difference between the paired two-samples (simulated and empirical) can not be rejected (at the significance level 5%). The 4th column reports the K-S test statistic (K-S) between the simulated sample and the regression line. Cases reported with an ‘*’ indicate that the 2 samples could come from the same distribution (at the significance level 5%). The 5th column indicates the correlation coefficient (r) between the simulated sample and the regression line.

The first scenario (Figure 6) is a simplistic model reflecting the experts' answers. General simulated patterns of depression are similar across each typology. Prevalence of depression is stable from retirement to the spouse's death. Then a notable peak occurs with a maximum reached after the elderly person has moved to another house. The prevalence then returns to a stable level. The patterns of depression prevalences using the answers of experts of Type 1 and Type 3, are higher than the one using the answers of experts of Type 2 throughout time. The simulated patterns using the answers of experts of Type 1, suggests a later but sharper onset of the peak, when the elderly person changes housing.

The simulated patterns using answers of experts of Types A and B indicate that both prevalences are initially at the same level. However, after a peak similar to the previous patterns, the prevalence of depression using Type B goes back to the initial level, while for Type A, the prevalence reaches a peak of higher amplitude and stays at a higher level.

The graphical comparison indicates a difference between the patterns of the empirical and simulated curves. This is confirmed by all statistical tests, showing a significant difference for all typologies of experts. Compared to other typologies, the simulation for experts of Type 2 provides the best fit, regarding the number of occurrences within the confidence interval.

In the second scenario (Figure 7), inducing the wedding and widowing processes, the behavior of the prevalence becomes increasing linear. The statistical tests show significant differences between the simulated and empirical curves. However, the similarity of the general trends with the slope of the regression line is remarkable, and this is confirmed by the high correlation coefficients in all cases. The simulated curves are above the regression line in all cases. The experts of Type 2 have a lower level than all other types providing again the best fit regarding the number of occurrences within the confidence interval. A late increase of prevalence in the last ages indicates the late depression of new widows having lost their spouse from natural death at 93.

In the third scenario (Figure 8), the effect of treatment induces a downwards translation of all curves, decreasing the overall prevalence levels of depression. The simulated curve of experts of Type 2 lies almost perfectly on the regression line, and the gap between the curves of the different typologies is reduced. The Wilcoxon Signed-Rank test shows no significant difference between the empirical and the simulated curves for experts of Type 2 and B. In addition, for these two types, the KS test shows no significant difference between the simulated curve and the regression line. The correlation coefficients are high in all cases.

In the fourth scenario (Figure 9), the distinction between the different socio-economic states amplifies the overall level of depression, as indicated by an upwards translation of all curves. Comparing the curves of both categories of SES independently showed that the disadvantaged population presents a higher level of depression (not shown here). The statistical tests show significant differences for all typologies but the good correlation coefficients are maintained.

Other simulations were run to test the relevance of parameters values and the combinations of different effects. The simulation with event ages fixed with null variation (stdv = 0) produced similar curves, indicating that this noise averages out between individuals and over all simulations. In the first scenario, modifying the Age Path by setting the age of widowhood at 61 or 75 produced better fit respectively with the apparent early and late maxima in the empirical prevalence. Treatment and socio-economic effects were also tested on the first scenario (no wedding), producing the same translation effects observed when considering wedding. Finally, a simulation was run mixing both treatment and socio-economic effects. The upwards and downwards translations of the curves almost cancelled each other out, and the curves were similar to the ones observed for scenario 2.

## Discussion

In this article, we demonstrate the application of Rule-Based Modeling as a tool to improve our understanding about the influence of social support and specific late-life events on depression among the elderly. The application and approach at hand may be used as a template for studying the epidemiology of other chronic conditions.

As we can see from the results, the fit between the simulated curves and the empirical data are not always optimal. This is often the case in simulation models used to study social phenomena. This link between the simulated and the empirical data remains a delicate topic that feeds the debate on the empirical validity of simulation models [40], [41].

However, two modeling traits need to be kept in mind while analyzing any kind of model. On the one hand, a model is a simplified representation of reality. We therefore assumed in our conceptual framework that the only drivers of elderly depression are socially related. If the patterns reproduced by the model do not correspond fully to observed patterns, this may indicate that “socially related rules” may not suffice, thereby establishing a need for further studies integrating additional drivers. On the other hand, a model is also a purposeful representation of reality. Hence the major aim of this model is not to produce a perfect representation of reality but to improve the conceptual understanding of the role of social factors related to late-life depression.

Taking this into consideration, a number of interesting insights emerge from our modeling exercise. Each individual scenario and their combinations provided useful lessons.

The first scenario assesses the experts' answers, independently of other effects. The simulated curves from this scenario indicate that a peak in the prevalence of depression is observed after the spouse's death. This could suggest that the experts attribute a large influence of the spouse on the Depressive Status. While this may appear as an intuitive truism, different answers from Types A and B experts indicate that this influence may be perceived differently. These two types of responses illustrate two facets of the perception of losing a spouse. For Type A experts, there was no further impact of spouses after their death, and this absence is not compensated by other sources of social support. For Type B experts, the spouse had a “ghost” contact influence after dying, though that influence was smaller than the influence of a living spouse.

The unique peak of depression in the simulated curve after the spouse's death does not fit the two apparent rises and falls in the empirical prevalence patterns. Changing the ages of widowhood actually allowed the simulated data to better fit with either one of these peaks. If the reason of these empirical peaks is only related to widowhood, this could possibly suggest that two waves of depression resulting from widowhood occur in the population at two different ages. For example, depressive people could die at younger ages, as already reported in some literature [42]. None of the scenarios included such a rule.

The second scenario includes a gender distinction and a widowhood effect. The results indicate a smooth increasing linear pattern through all typologies of experts. This is the result of the spread of the year of widowhood depression through the late-life span. Furthermore, this year of depression induces the use of the depressive Contact Table, two years after the death of the spouse. Thus, the widow is not only depressed during one year, but this year of bereavement can also modify the social landscape, resulting in a possible longer-term depressive vicious cycle. This effect has been observed before and already encouraged the modification of urban structures of widow neighborhoods together with initiatives for new social engagements for widows [11].

In these two first scenarios, the levels of depression prevalence in the simulations appear higher than the levels obtained from empirical data. On the one hand, this could indicate that respondents had a tendency to quantify social parameters overestimating the prevalence of depression in the elderly. It is difficult to determine whether this could be due to an unconscious perception (“stereotype”) of the experts that “old age is depressing” or whether our conceptual framework does not optimally reflect the experts' internal representations of late-life depression. However, the overestimation of elderly depression itself has already been debated [43] and may explain a possible overprescription of antidepressants to the elderly [44]. On the other hand, this higher level of simulated prevalences could as well be explained by an underestimation of elderly depression by the empirical data. Indeed, symptoms of depression may be atypical and stay unnoticed [45]. Moreover, denial and the stigma of diagnosis can further hamper the accurate assessment of depression in the elderly [46].

In the third scenario, the effect of a treatment by psychotherapy, as modeled, decreases the overall level of prevalences of depression compared to the second scenario. While the indicator of depression used did not rely on treatment or diagnostic information, the reported empirical prevalences include inherently people under treatment. However no treatment effect was mentioned in the questionnaire. Hence, this result could suggest that experts evaluated correctly the natural prevalence evolution of depression (second scenario), independently of the positive effect of a treatment (induced in the third scenario). Considering the statistical tests, this scenario provides the best fit with the empirical patterns.

In the fourth scenario, the conception of one simple rule structured our inquiries about the correlation between the socio-economic status of elderly people and their depressive status. When investigated independently, the curves of each SES categories are parallel, at different levels. This is due to the fact that each individual is assigned an SES permanently, and the negative effect is then permanent, as modeled. In fact, each individual could also perceive a personal dynamical change (worsening) of SES through the late-life time. In fact, the “relative” evolution can also have an impact on the depressive status at all ages [47]. To some extent, a relative change in the social status is already embedded within our initial conceptual framework, as the different life events modify the social landscape. Regarding the economic status, the income level was chosen naively as a simple criterion to classify groups. However this distinction might not be appropriate as health disparities by income are reported to be reduced at older ages [48]. In fact, people surviving into old age might be skewed towards economic advantage [49].

Finally, in the three last scenarios, the good correlation between the trends of simulated curves and the slope of the empirical regression line is remarkable. This result could suggest that, through our conceptual framework, experts may have a good internal representation of the factors dynamics influencing the depression through the late-life time. Further, the indices in Table 1 suggests that the results of the simulation based on the answers of Type 2 experts provide the best fit, through all scenarios. Type 2 experts are health care professionals and have frequent contact with elderly persons. Hence, it appears consistent with common sense that their better-informed knowledge enables them to adapt their opinions within our abstract analogy. This may suggest that our conceptual framework, combined with rules mimicking real population evolution, together with the opinions of well-selected experts, can reproduce a realistic evolution of the prevalences of depression. Further interviews with experts of all types could help to explain the sources of discrepancies and similarities between expert types. In addition, this exploration could raise additional questions and encourage other possible conceptual frameworks.

Several choices made in the development of our model may show some shortcomings. For example, we used data from repeated cross-sectional studies. This may introduce several biases (e.g. possible confusion between age, period, and cohort effects) that could be avoided in longitudinal studies. The use of such classical longitudinal data in future work may improve our model. However, currently available cohort studies (e.g. the Share cohort study and the Annual Belgian Household Panel Survey) do not cover social and life-course mechanisms that are important to this study.

Moreover, the design of the rules related to the widowhood effect, the treatment effect and the socio-economic distinction can be criticized. However, the purpose was to prove the experimental capacity of RBM. This simple model can actually be used to structure these criticisms and further refine the model.

In addition, our model is purposefully simplistic when using the concept of depression. Indeed, our focus was not on describing precisely the pathology, but to study its social aspects and the experts' perceptions. Using the word “depression” in our questionnaire may have biased the answers, suggesting a self-evident association between elderly people and depression [14], [50]. In place of depression, using the concepts of mood, such as happiness or sadness might have been more neutral and would not have conflicted with our illustrative purposes.

In conclusion, this paper indicates that RBM provides a possible balance between the technical thoroughness of quantitative studies, and the exploratory power of qualitative studies. The qualitative aspects can help to reveal complex mechanisms and abstract analogies

The simulation model presented here should be seen primarily as a tool for thinking and learning [41]. Indeed, the comparison between the simulated and the empirical data enlightens different facets of our personal internal representation of the topic at hand. In addition, it helps to identify and properly formulate new context-specific questions [51] submitted to new assumptions. However, causal explanations and the predictive power of the simulation model should be limited and interpreted in the light of these same contexts and assumptions, as this is the case in statistical analysis.

Hence, the designed analogies or conceptual framework can be investigated and tested as in an experimental lab [52], without long and costly longitudinal studies. In fact, this process can assist in the design of new models, which may better guide the collection of new data [51].

Another advantage of RBM lies in its flexibility. Virtually all scenarios can be simulated, with as many interactions and variables as needed, allowing additional rules to be included. However, the abstraction and the rules should always be kept simple enough to be understood, and logical enough to allow the learning process to continue [53].

This flexibility and the formalism of RBM make it a powerful tool of communication and negotiation between experts, providing an interactive medium for social exploration [54]. As the simulation program requires a formal language with simple rules, it forces experts to build theories following an architecture that reduces ambiguity. The informative interactions between modelers and experts allow for improving and learning from the elicitation in the absence of empirical data. Simulated data can fuel further discussion among experts, and new abstractions can be considered, forming a dynamic learning cycle of trial-error-rework.

RBM does not require high-level skill in mathematics, and may attract researchers from quantitative and qualitative backgrounds alike. Combining wisely both insights can only increase our understanding of complex phenomena.

## Supporting Information

Appendix S1 **Questionnaires as presented to the experts (EN).**
(XLS)

Appendix S2 **Questionnaires as presented to the experts (FR).**
(XLS)

Appendix S3 **Mean Contact and Impact Tables.**
(DOC)

Appendix S4 **Consensus measures for Contact and Impact Tables.**
(DOC)

Appendix S5 **Empirical data.**
(DOC)

Appendix S6 **Source codes (see also** https://sourceforge.net/projects/rbmelddep/ **).**
(TXT)

Appendix S7 **Installation and user instructions.**
(DOC)
