# Supplementary material for: Rule-Based Modeling of Chronic Disease Epidemiology: Elderly Depression as an Illustration
Source: PLoS One. 2012 Aug 28;7(8):e41452. doi: 10.1371/journal.pone.0041452 (PMC3429481; doi:10.1371/journal.pone.0041452)
Supplement: Appendix S3 — Mean Contact and Impact Tables. (DOC) [file pone.0041452.s003.doc]

APPENDIX 2 : MEAN CONTACT AND IMPACT TABLES

**Appendix 2.1 :** Contact table quantifying the relative importance (in %) of each social actor at each key late-life event for a **non-depressive** elderly. Answers are averaged over all experts matching the typology definitions.

**Appendix 2.2 :** Contact table quantifying the relative importance (in %) of each social actor at each key late-life event for a **depressive** elderly. Answers are averaged over all experts matching the typology definitions.

**Appendix 2.3 :** Impact table quantifying the probabilities of positive, negative, or neutral impacts resulting from contacts with the social actors. Answers averaged over all experts matching the typology definitions.
