# Supplementary material for: Rule-Based Modeling of Chronic Disease Epidemiology: Elderly Depression as an Illustration
Source: PLoS One. 2012 Aug 28;7(8):e41452. doi: 10.1371/journal.pone.0041452 (PMC3429481; doi:10.1371/journal.pone.0041452)
Supplement: Appendix S4 — Consensus measures for Contact and Impact Tables. (DOC) [file pone.0041452.s004.doc]

APPENDIX 4 : CONSENSUS MEASURES FOR

CONTACT AND IMPACT TABLES

***1) Interquartile Range***

**Appendix 4.1 :** Interquartile Range of expert's answers within the Contact Table for the non-depressive case. Experts are classified following the typology definitions.

**Appendix 4.2 :** Interquartile Range of expert's answers within the Contact Table for the depressive case. Experts are classified following the typology definitions.

**Appendix 4.3 :** Interquartile Range of expert's answers within the Impact table. Experts are classified following the typology definitions.

1. ***Percentile 25 and percentile75***

**Appendix 4.4 :** Percentile 25 -Percentile 75 of expert's answers within the Contact Table for the non-depressive case. Experts are classified following the typology definitions.

**Appendix 4.5 :** Percentile2 5 -Percentile 75 of expert's answers within the Contact Table for the depressive case. Experts are classified following the typology definitions.

**Appendix 4.6 :** Percentile 25 -Percentile 75 of expert's answers within the Impact table. Experts are classified following the typology definitions.
