# Supplementary material for: Rule-Based Modeling of Chronic Disease Epidemiology: Elderly Depression as an Illustration
Source: PLoS One. 2012 Aug 28;7(8):e41452. doi: 10.1371/journal.pone.0041452 (PMC3429481; doi:10.1371/journal.pone.0041452)
Supplement: Appendix S5 — Empirical data. (DOC) [file pone.0041452.s005.doc]

APPENDIX 6 : Empirical data used

# Note on HIS data

*“The results that are presented are weighted percentages or means, and their 95% confidence intervals (CI), together with the total number of respondents (N is unweighted). The use of weighting factors adjusts for differences between the survey sample and the real population, in terms of the distribution by age, sex, size of the household and province. By weighting the data the results are representative for the total population, at national, regional and provincial level.”* [*https://www.wiv-isp.be/epidemio/hisia/data.htm*](https://www.wiv-isp.be/epidemio/hisia/data.htm)

# 1) Empirical prevalences by age

# 2) Rate depressive men and women at 61

***Appendix 2.2.a:*** *From HIS data base: Percentages of depressed respondents and total number of respondent of the population of 61 years old distinguised by gender fro 2001-2004-2008. https://www.wiv-isp.be/epidemio/hisia/index.htm*

***Appendix 2.2.b:*** *Number of Depressed respondents are computed by year , summed and rounded to retrieve the percentage of depressed men and women at 61.*

# 3) Rate married women at 65

Rate married women=34769/ (34769+16565)=67.73%

***Appendix 3 :*** *From EuroStat* *Rate of married women computed from the Belgian household composition in 2001-2004-2008 For the purpose of the simulation Widow and divorced at 65 were considered as singles. http://epp.eurostat.ec.europa.eu/portal/page/portal/population/data/database*

# 4) Ratio of men and women at 65

Rate of women=186/333=55.8%

***Appendix 5:*** *From HIS data base: Number of respondents of 65 years old distinguished by gender.* [*https://www.wiv-isp.be/epidemio/hisia/index.htm*](https://www.wiv-isp.be/epidemio/hisia/index.htm)

# 5) Rate of elderly persons treated for depression by psychotherapy

***Appendix 5:*** *From HIS data base: Percentages of depressed respondents between 65-92 with a reported depression in the past 12 months and that engaged in a psychotherapy for this problem. Only year 2004 and 2008 were available.* [*https://www.wiv-isp.be/epidemio/hisia/index.htm*](https://www.wiv-isp.be/epidemio/hisia/index.htm)

# ***6) Rate of disadvantaged elderly persons at 65***

*Percentage of elderly people with income <750*€ =613/5313 = 11.53

***Appendix 6:*** *From HIS data base: Number of respondents by level of income 65 in 2001-2004-2008.* [*https://www.wiv-isp.be/epidemio/hisia/index.htm*](https://www.wiv-isp.be/epidemio/hisia/index.htm)

# ***7) Mortality Rates***

***Appendix 7:*** *From Belgian Federal Economy Service: Mortality Rate computed over the population size (px) and number of deceased (dx) in 2001-2004-2008.*
