# Supplementary material for: Rule-Based Modeling of Chronic Disease Epidemiology: Elderly Depression as an Illustration
Source: PLoS One. 2012 Aug 28;7(8):e41452. doi: 10.1371/journal.pone.0041452 (PMC3429481; doi:10.1371/journal.pone.0041452)
Supplement: Appendix S7 — Installation and user instructions. (DOC) [file pone.0041452.s007.doc]

**Rule-Based Model of Chronic Disease Epidemiology:**

**Elderly Depression as an Illustration**

**Appendix S7: Installation and Users Instructions**

*Chiem Jean-Christophe*

*Jean Macq*

*Niko Speybroeck*

Institute of Health and Society (IRSS)

Université Catholique de Louvain

The Matlab files described here refers the simulation described in the paper :

“Rule-Based Model of Chronic Disease Epidemiology: Elderly Depression as an Illustration”

published in Plos One.

The m.files can be downloaded using the following link: https://sourceforge.net/projects/rbmelddep/.

# 1) Installation:

The package with the source code are made of 9 .m files.

- **param_TypeAll.m** : loading the Mean Contact and Impact Tables of All experts
- **param_Type1.m** : loading the Mean Contact and Impact Tables of experts of Type 1
- **param_Type2.m** : loading the Mean Contact and Impact Tables of experts of Type 2
- **param_Type3.m** : loading the Mean Contact and Impact Tables of experts of Type 3
- **param_TypeA.m** : loading the Mean Contact and Impact Tables of experts of Type A
- **param_TypeB.m** : loading the Mean Contact and Impact Tables of experts of Type B
- **parameters.m**: file where you can modify the parameters values, decide of scenarios, and decide what typology of expert you want to use.
- **Simulation_RBM_eld_dep_no_wed.m**: simulation considering no gender distinction and no wedding procedure
- **Simulation_RBM_eld_dep.m**: simulation considering the gender distinction and a wedding procedure


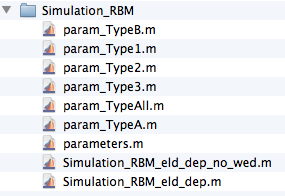


A distinction between the file regarding the gender and the wedding was done to enhance the readability of the code. However the 2 codes are very similar.

The files should be installed in the same folder. **Only the parameters.m file should be modified by the user.**

# 2) Parameters and scenarios


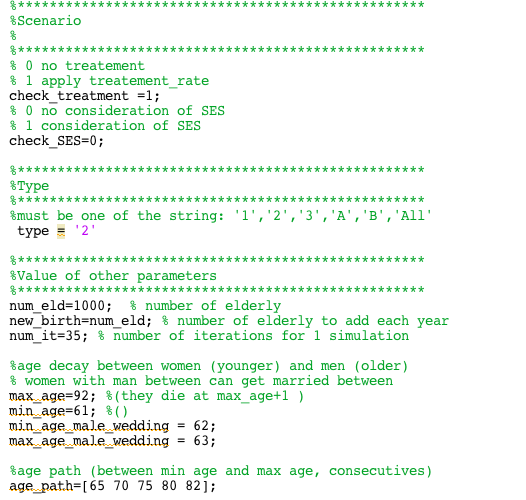


The parameters.m files begins by a declaration of the parameters as global variables which will be used throughout the algorithm. After this declaration, the values of these parameters can be modified. The scenario definition consists in checking the variables check_treatment and check_SES (0: disabled , 1: enabled) .

Together with the distinction of the algorithms (Simulation_RBM_eld_dep_no_wed.m and Simulation_RBM_eld_dep), the 4 scenarios discussed in the paper can be simulated using the parameters as follows:

Other combinations can be tested. The values of other parameters may also be modified, but refer to the comments in the code for the expected values. For examples, the value in the age_path must be between min_age and max_age and be consecutive.

# 3) Simulation Inputs and Outputs

The generic function definitions of both simulations functions are:

myprev_m1= Simulation_RBM_eld_dep_no_wed(WS_list, WS_dir, prefix)

myprev_m1= Simulation_RBM_eld_dep(WS_list, WS_dir, prefix)

The following inputs needs to be entered

**WS_list:** list of indices represents the number of times the simulation should be run.

ex: to run the simulation 50 enter the list 1:50

**WS_dir:** directory where to save the .mat files generated for each simulation

**Prefix:** Prefix of the name of the .mat files generated for each simulation

(Remind that the parameters of the simulation are entered in parameters.m)

Several outputs are generated. For each simulation a Workspace with all the relevant information is saved in 'WSdir' with name **'Prefix_typex_y.mat'** where x refers to the typology of experts and y the number of iterations. This workspace records:

- values of the parameters, Contact and Impact tables

- scenario info (check_treatment, check_SES)

- prevalences of the last iterations of the simulation (myprev_m1)

- indices of fit computes by functions compute_TS and compute_Num_index:

- other variables are only technique variables

For each simulation the prevalences of the current simulation are saved and accumulated in variable myprev_m1.

At the end of all the simulation the saved prevalences are then averaged and the mean is drawn together with the empirical prevalences retrieved from The online Belgian Health Interview Survey (HIS) and the associated regression line.

An array named fit_index is generated with the numerical and statistical indices of fitness. The following indices are computed:

1) The **number of occurrences** of the mean of the simulated curves falling within the CI bounds (between 65 and 92)

2-3) The **Wilcoxon signed rank test** [P,H] for zero median two-sided test of the hypothesis that the difference between the matched samples in the vectors X and Y comes from a distribution whose median is zero:

- P is the probability of observing the given result, or one more extreme, by chance if the null hypothesis ("median is zero") is true.

- H=0 indicates that the null hypothesis ("median is zero") cannot be rejected at the 5% level. H=1 indicates that the null hypothesis can be rejected at the5% level.

4-6) The 2 **samples Kolmogorov-Smirnov test** [H,P,KSSTAT] between the simulated curve and the regression line, testing whether the 2 samples could come from the same distribution.

- H = 0 => Do not reject the null hypothesis at significance level 5%. H = 1 => Reject the null hypothesis at significance level 5%.

- P=asymptotic P-value

- KSSTAT= test statistic

7) The **correlation coefficient** between the simulated curve and the regression line

# 4) Example

The ***Scenario 3*** described in the manuscript can be implemented as follows. In the ***parameters.m,*** the different parameters should be set as indicated in the figure below. Hence the simulation will enable the treatment effect, and it will use the answers of experts of Type 2 for the Contact and Impact Tables. The population will count 1000 elderly persons at 65 and 35 iterations will be run by simulation.


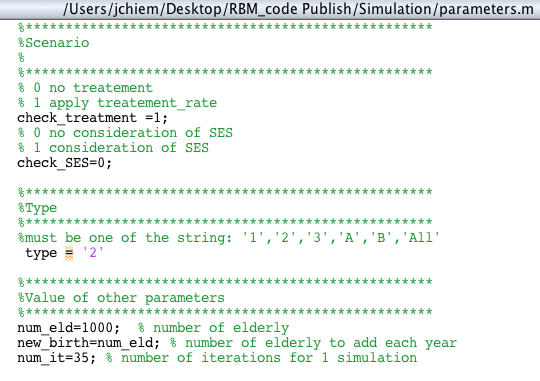


To consider the gender distinction and a wedding process, the ***Simulation_RBM_eld_dep.m*** should be run. To run 50 simulations and save your 50 workspaces in the directory '/Users/Desktop/WS_test' with prefix of name 'My_simulation', use the following command:

[myprev_m1]= Simulation_RBM_eld_dep(1:50, '/Users/Desktop/WS_test','My_simulation');


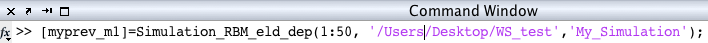


The current simulation and iteration are displayed continuously, to indicate the simulation is running. This is illustrated in the next figure for the 31^st^ simulation between the 15^th^ and 20^th^ iterations.


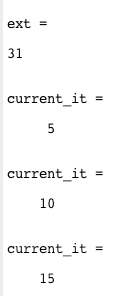


At the end of the simulation a graph is displayed with the empirical data, the regression line and the simulated pattern.


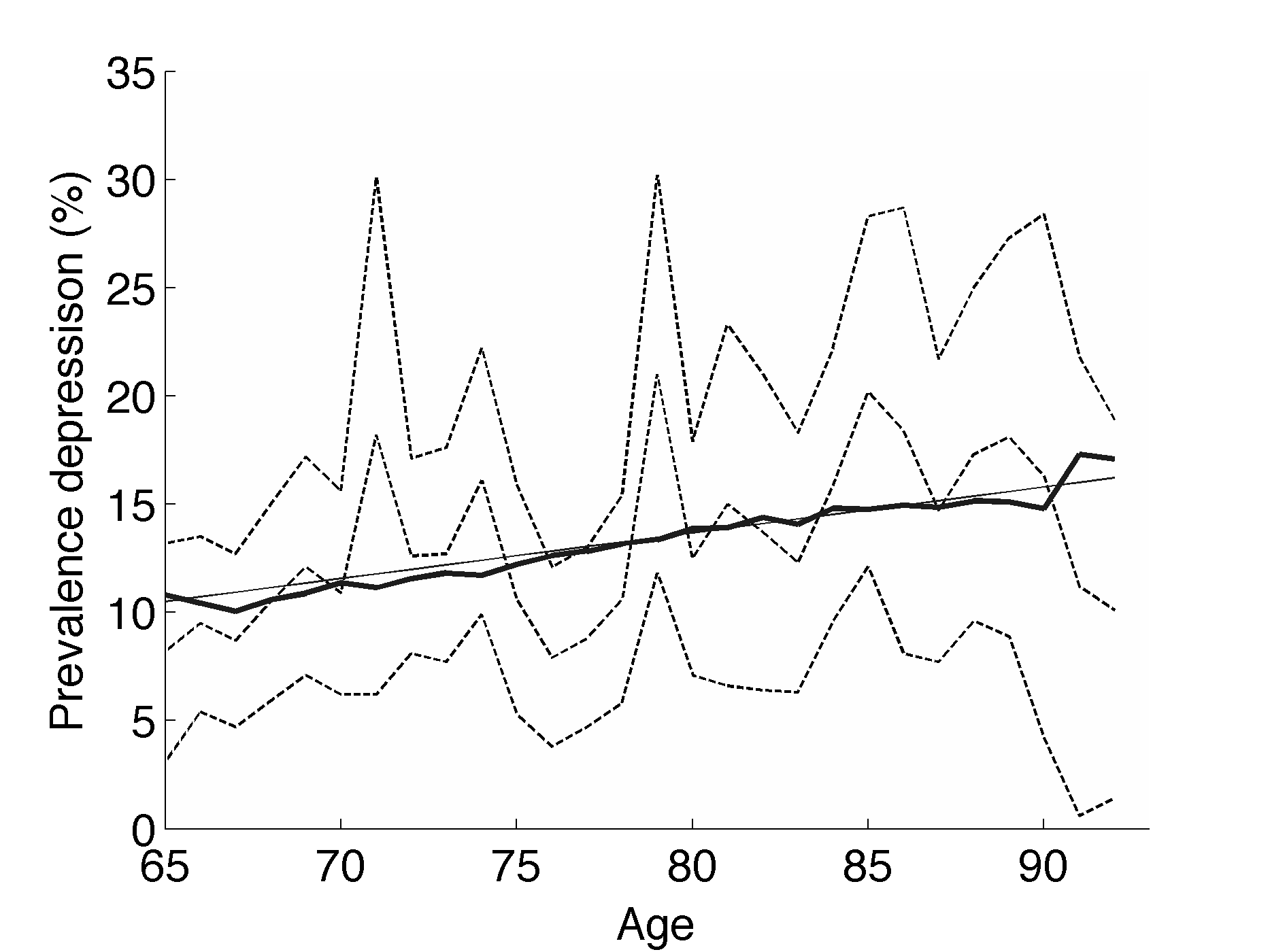
Fifty workspaces have been saved in the folder 'Users/desktop/WS_test' and named 'My_Simulation_type2_x' with x going from 1 to 50:


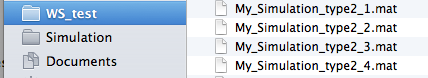


In the command window the variable fit_index is displayed as follows.


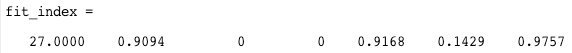
This sequence of indices can be described as follows:

- 27 points of the simulated curves falls withing the CI
- 0.9094 is the probability of observing the given result, by chance if the median of the difference between the two samples is true
- 0 indicates that the hypothesis that the median is zero, can not be rejected
- 0 indicates that the hypothesis that the 2 samples can come from the same distributions can not be rejected
- 0.9168 is the value of the corresponding asymptotic p-value
- 0.1429 is the value of the K-S statistic
- 0.9757 is the correlation coefficient.

These results are similar to those reported in Table 1 for Scenario 3 of the manuscript. This example was run on a Macbook Pro with Mac Os X Version 10.7.3. Twenty-nine minutes were required for its completion.
